# Young children’s voices in an unlocked Sweden during the COVID-19 pandemic

**DOI:** 10.1177/14034948221108250

**Published:** 2022-07-07

**Authors:** M. Jenholt Nolbris, S. Ragnarsson, A.-L. Brorsson, M. Garcia de Avila, M. Forsner, I. Kull, A. L. Olinder, J. Mattson, S. Nilsson, A.-C. Rullander, L.-L. Rydström, P. Olaya-Contreras, M. Berghammer

**Affiliations:** 1 Institute of Health and Care Sciences, University of Gothenburg, Sweden; 2 The Queen Silivia Children’s Hospital Sahlgrenska University Hospital, Sweden; 3 Department of Epidemiology and Global Health, Umeå University, Sweden; 4 Department of Neurobiology, Care Sciences and Society, Karolinska Institutet, Sweden; 5 Department of Nursing, Botucatu Medical School - UNESP, Brazil; 6 Department of Nursing, Umeå University, Sweden; 7 Department of Biosciences and Nutrition, Karolinska Institutet, Sweden; 8 Department of Clinical Science and Education, Karolinska Institutet, Sweden; 9 Sachs’ Children and Youth’s Hospital, Södersjukhuset, Sweden; 10 Department of Health Science, The Swedish Red Cross University College, Sweden; 11 Department of Learning, Informatics, Management and Ethics, Karolinska Institutet, Sweden; 12 Centre for Person-Centred Care, University of Gothenburg, Sweden; 13 Department of Health Sciences, University West, Sweden

**Keywords:** Anxiety, children, COVID-19 pandemic, mixed-methods, online survey, Sweden

## Abstract

**Aims:**

During the COVID-19 pandemic, Sweden was one of the few countries that rejected lockdowns in favour of recommendations for restrictions, including careful hand hygiene and social distancing. Preschools and primary schools remained open. Several studies have shown negative impacts of the pandemic on children, particularly high levels of anxiety. The study aim was to explore how Swedish school-aged children aged 6–14 years, experienced the COVID-19 pandemic and their perceived anxiety.

**Methods:**

In total, 774 children aged 6–14 years and their guardians answered an online questionnaire containing 24 questions, along with two instruments measuring anxiety: the Children’s Anxiety Questionnaire and the Numerical Rating Scale. A convergent parallel mixed-methods design was used for analysing the quantitative and qualitative data. Each data source was first analysed separately, followed by a merged interpretative analysis.

**Results:**

The results showed generally low levels of anxiety, with no significant sex differences. Children who refrained from normal social activities or group activities (*n*=377) had significantly higher levels of anxiety. Most of the children were able to appreciate the bright side of life, despite the social distancing and refraining from activities, which prevented them from meeting and hugging their loved ones.

**Conclusions:**

**These Swedish children generally experienced low levels of anxiety, except those who refrained from social activities. Life was nonetheless mostly experienced as normal, largely because schools remained open. Keeping life as normal as possible could be one important factor in preventing higher anxiety and depression levels in children during a pandemic.**

## Background

Sweden was one of few countries that did not have a lockdown during the COVID-19 pandemic; recommendations for thorough hand hygiene and restrictions such as social distancing were initiated. This meant limiting close contact with people you do not live with, both indoors and outdoors [1]. Preschools and primary schools have also remained open throughout the pandemic, to prevent adverse effects such as loss of learning opportunities and a negative impact on children’s mental and physical health. Children in general were not found to become severely ill with a COVID-19 infection [1]. When the World Health Organization (WHO) classified the outbreak of the coronavirus disease COVID-19 as a pandemic on 11 March 2020, more than 200 countries decided to lock down large parts of their society in an attempt to curb the spread of the infection. Many researchers have investigated how the pandemic has affected different adult populations, and in an earlier review, increased levels of post-traumatic stress syndrome and depression following infection with the COVID-19 virus were reported [2].

Historically, children in all societies have been severely affected by epidemic diseases. By the middle of the 20th century, polio epidemics were widespread around the world, causing early death or lifelong paralysis, but today the disease is on the verge of extinction, thanks to vaccinations [3]. In 2009, swine flu (the influenza A virus H1N1) spread around the world, particularly affecting children and young people, and schools were then closed in many countries to reduce the spread of infection [4]. Beyond purely medical research, there is a lack of studies highlighting children’s perspectives on these past epidemics. Thus, it is important to evaluate how children experience the COVID-19 pandemic.

In a recently published study from Brazil, participating children expressed being more worried during the ongoing pandemic than during normal conditions [5]. In a large Swedish study, where 1700 adolescents aged 15–19 years responded to an online survey, the adolescents reported being compliant with rules and regulations but at the cost of their psychosocial functioning. They also experienced poorer mental health than before the pandemic [6]. According to the WHO, children living in socioeconomically disadvantaged areas have been reported to be particularly exposed to lockdowns due to the COVID-19 pandemic [5,7–9]. Since there was no lockdown in Sweden but only certain restrictions, it is of interest to see how this has affected children aged 6–14 years.

## Aim

The study aim was to explore how a convenience sample of Swedish schoolchildren, aged 6–14 years, experienced the COVID-19 pandemic and their perceived anxiety.

## Methods

### Study design

A convergent parallel mixed-methods design [10] was chosen, where quantitative and qualitative data were collected at the same time but analysed separately and then merged, leading to a combined result.

The quantitative research questions:

- Has the experience of refraining from social activities during the COVID-19 pandemic affected children’s perceived anxiety?
- Have sociodemographic factors influenced the relation between refraining from social activities and children’s anxiety during the COVID-19 pandemic?

The qualitative research question:

- What are children’s thoughts about their situation during the COVID-19 pandemic?

### Participants

In total, 774 children participated in the study (Table I). The inclusion criterion was children aged 6–14 years, and they participated together with their guardians. The survey was sent to the guardians, who gave their written consent to participate as well as the child.

**Table I.** Descriptive data for all included children (*n*=774) in the study and for the subgroup included in the qualitative analysis (*n*=151).

	Whole group		Subgroup included in qualitative analysis	
Characteristics	Total	Breakdown *n* (%)	Total	Breakdown *n* (%)
Sex	774		151	
Boys		368 (47.5)		62 (41.1)
Girls		405 (52.3)		89 (58.9)
Age (years)	768		150	
6–9		410 (53.0)		88 (58.7)
10–12		228 (29.5)		46 (30.6)
13–14		130 (16.8)		16 (10.6)
Chronic diseases /disabilities	772		151	
Yes		74 (9.6)		18 (11.9)
No		698 (90.2)		133 (88.1)
Community size (inhabitants)	772		150	
>500,000		88 (11.4)		14 (9.3)
100,000–499,999		461 (59.7)		92 (61.3)
<100,000		223 (28.9)		44 (29.3)
Household size	606		120	
1–3		516 (85.1)		104 (86.7)
4–7		87 (14.4)		16 (13.3)
> 8		3 (0.5)		-
Level of education (guardian)	752		146	
Lower than university		166 (22.1)		21 (14.4)
University		586 (77.9)		125 (85.6)
Employment (guardian)	763		150	
Working		666 (88.6)		131 (87.3)
Sick leave and other		41 (5.4)		11 (7.3)
Studying		25 (3.3)		5 (3.3)
Unemployed		19 (2.7)		3 (2.0)
Reduced income during the pandemic (guardian)	764		151	
Yes		111 (14.5)		26 (17.2)
No		654 (85.5)		125 (82.2)
Attended school during pandemic (child)	774		151	
Yes		752 (97.2)		142 (94.0)
No		22 (2.8)		9 (6.0)
Distance education during pandemic (child)	774		151	
Yes (full or partial)		79 (10.2)		19 (12.6)
No		695 (89.8)		132 (87.4)
Anyone in the family has had COVID-19			148	
Yes		55 (7.4)		10 (6.8)
No		533 (71.5)		101 (68.2)
Do not know		157 (21.1)		37 (25.0)
Social group activities / sports (child)	771		151	
Refrains from social group activities / sports		389 (50.3)		95 (62.9)
Participates in social group activities / sports		382 (49.4)		56 (37.1)

### Data collection

An online survey was distributed between 7 July–8 November 2020 using the web platform esMakerNX3, version 3.0 (Entergate AB, Halmstad, Sweden). A convenience sampling method through snowballing was used, in which the Web survey was distributed by the research group and their social network contacts and posted on social media, primarily through Facebook and Instagram. The survey was also sent to primary schools within the researchers’ network, mainly across three counties of Sweden, for help with the distribution. It took approximately 5–10 min to fill out the survey. The guardians answered questions 1–18 and the children answered questions 19–24 themselves or with the guardian’s help if needed.

### The questionnaire

The questionnaire, which consists of 24 items, is based on a questionnaire developed and used in a study in Brazil investigating the prevalence of anxiety among children during the COVID-19 pandemic [5]. The Swedish-adapted version of the questionnaire was first tested in a pilot study with 33 participants, where 20 participants were aged 6–14 years and 13 participants were aged 15–19 years. After the pilot study, some adjustments were made to further clarify the questions. The pilot test data were not included in the main data collection.

The questionnaire included demographic questions on residency (housing and community size), household size, education level and employment status of the guardian, the age and sex of the child and whether the child had chronic diseases or disabilities. In addition, there were questions related to the pandemic: whether anyone in the family had had COVID-19, whether the guardian’s monthly income had decreased during the pandemic, whether the child had attended school or had distance education and to what extent the child refrained from social activities. One open question was included: ‘Is there something you would like to add?’, allowing the children to express their own thoughts in relation to the COVID-19 pandemic.

To measure level of anxiety, two visual scales were used, the Children’s Anxiety Questionnaire (CAQ) [11], consisting of four pictorial items showing facial expressions, each representing a different type of emotion. The CAQ scores ranged from 4–12 points, with 4 points signifying low anxiety and 12 points signifying the highest level of anxiety. CAQ scores above 9 points are classified as intense anxiety [5]. The second scale was the Numerical Rating Scale (NRS), an 11-point scale indicating their current anxiety. The NRS score ranges from 0–10 levels, where 0=‘calm’ and 10=‘very anxious’ [12,13].

### Quantitative analyses

Statistical analyses were conducted using The Statistical Package for the Social Sciences (SPSS) versions 25.0 and 27.0 for Windows (IBM Corp., Armonk, New York, USA). The prevalence of intense anxiety was measured by the CAQ and NRS and the differences between categories was tested using the Pearson’s chi-squared test. The difference between the children reporting intense anxiety and those with lower anxiety levels on the pandemic-related variables (contextual and individual factors) were tested with the independent samples *t*-test. Correlations between the independent variables were tested with Spearman’s rank correlation coefficient and no correlations exceeded 0.13; thus, the assumption of multicollinearity among the independent variables was rejected. The residuals of the dependent variable (CAQ) were treated as normally distributed due to the rather large sample size and levels of skewness (0.99) and kurtosis (0.97) [14]. A general linear model (GLM) was used to analyse the association between refraining from social activities and perceived anxiety.

Perceived anxiety (CAQ score) was used as the dependent variable in the GLM analyses. The independent variable *Refrains from social activities* had three response alternatives: 1 ‘totally’, 2 ‘partly’ and 3 ‘not at all’, which were dichotomised into ‘yes’ for alternatives 1 and 2 and ‘no’ for alternative 3. The GLM crude model (Model 1) tested associations between the variable *Refrains from social activities* and perceived anxiety. Thereafter in Models 2–4, the covariates sex, age, chronic disabilities and reduced income were added stepwise to the regression model. The significance level was set to *p*<0.05.

### Qualitative analyses

There were 326 answers to the open question ‘Is there something you would like to add?’. Of these, 160 children answered ‘no’ and were not analysed further. Answers which were considered to originate from the guardian were excluded (*n*=15). This resulted in 151 answers ranging from a few words to longer responses with several sentences. The answers were read several times and were subjected to an inductive content analysis according to Elo and Kyngäs [15], in which words or phrases sharing a common meaning (meaning units), are distilled into content-related categories.

### Ethical aspects

Ethical approval was obtained from the Swedish Ethical Review Authority (ref. 2020-02547) and the participants received information about the study in a separate part of the online survey. The survey was anonymous.

## Results

The total study population consisted of 774 children aged 6–14 years (mean age 9.5 years), with a higher proportion of girls than boys (52.5% vs 47.5%). The participating guardians were mainly mothers (83.4%). Most of the participants lived in cities with less than 500,000 inhabitants (88.6%), and a high proportion of the guardians had a university degree (77.9%). Most of the children had not had distance education during the pandemic (89.8%) (Table I).

### Quantitative results

**Figure 1.**
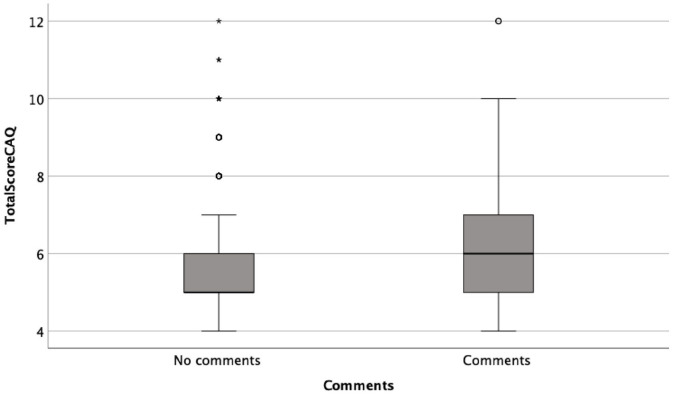
Levels of anxiety measured of anxiety measured with the Children’s Anxiety Questionnaire (CAQ) total score in the group who answered the open question (Comments) and the group who did not (No comments). The minimum total score=4, representing the lowest anxiety level on all four items.

All 774 children were included in the quantitative analysis. The level of perceived anxiety was low, both when measured with the NRS spanning 0–10 (mean 2.4, standard deviation (SD) 2.3, median 2) and when measured with the CAQ scale spanning 4–12 (mean 5.8, SD 1.5, median 5). There were no significant differences in anxiety between boys and girls (Table II). The correlation between NRS and CAQ was 0.54 (*p*<0.001). The level of anxiety was significantly higher in the subgroup answering the open question (*n*=151), versus the 623 who gave no answer, CAQ 6.07 versus 5.68, respectively (*p*=0.016) (Figure 1) and NRS 2.87 versus 2.3, respectively (*p*=0.017). Only refrains from social activities and reduced income showed a statistically significant correlation with perceived anxiety measured with CAQ (Table II). Accordingly, these factors, together with sex and age (6–9, 10–12 and 13–14 years) were included as covariates in the GLM model (Table III).

**Table II.** Characteristics of children who reported intense anxiety on the Children’s Anxiety Questionnaire (CAQ) or the Numerical Rating Scale (NRS).

		**CAQ score >9** 19/749 (2.4%)			**NRS score >7** 20/745 (2.6%)	
	Total	*n* (%)	*p* ^a^	Total	*n* (%)	*p* ^a^
**Sex**						
Boys	353	8 (2.3)		350	7 (2.0)	
Girls	395	11 (2.8)	0.892	394	13 (3.3)	0.542
**Age**						
6–9 years	400	13 (3.3)		396	12 (3.0)	
10–12 years	221	3 (1.4)		220	5 (2.3)	
13–14 years	124	3 (2.4)	0.357	124	3 (2.4)	0.838
**Chronic disease/disabilities**						
Yes	72	3 (4.2)		71	4 (5.6)	
No	675	16 (2.4)	0.357	672	16 (2.4)	0.107
**Reduced income during the pandemic**						
Yes	106	7 (6.6)		110	5 (4.5)	
No	635	12 (1.9)	0.004^b^	627	15 (2.4)	0.200
**Refrains from social activities**						
Yes	377	17 (4.5)		376	15 (4.0)	
No	370	2 (0.5)	0.001^b^	367	5 (1.4)	0.027^b^

a Chi-square test; ^b^significant at *p*<0.05.

**Table III.** Differences in the dependent variable, children’s perceived anxiety, measured by Children’s Anxiety Questionnaire (CAQ), in relation to refraining from social activities during the COVID-19 pandemic, stepwise adjusted for demographic and pandemic related factors.

		Model 1		Model 2		Model 3		Model 4
	*n*	B(SE) crude	*n*	B(SE)adj¹	*n*	B(SE)adj²	*n*	B(SE)adj³
Refrains from social activities								
Yes	377	Ref	376	Ref	375	Ref	374	Ref
No	370	−0.57 (0.11)^a^	367	−0.57 (0.11)^a^	367	−0.55 (0.11)^a^	361	−0.52 (0.11)^a^
¹Age				ns		ns		ns
¹Sex				ns		ns		ns
²Chronic diseases/disability						^a^		^a^
³Reduced income during pandemic								^a^
Adj R²		0.035		0.034		0.057		0.060

Adj: adjusted; B: beta coefficient; SE: standard error.

a *p*<0.05.

The prevalence of children with intense anxiety (CAQ score >9 or NRS score >7) in the total study population was 2.5% (CAQ score) and 2.7% (NRS score) (Table II). Considering the factors included in the regression models (the child’s sex, age, chronic disease/disabilities and reduced parental income during the pandemic), the prevalence of intense anxiety measured by CAQ was significantly higher among children who refrained from social activities compared to children who did not (4.5% vs 0.5%, *p*=0.001). The pattern was almost the same for children reporting intense anxiety on NRS (Table II). Children whose parents had a reduced income due to the pandemic showed a significantly higher prevalence of intense anxiety on CAQ in contrast to children whose parents’ income was not affected by the pandemic (6.6% vs 1.9%, *p*=0.004).

#### Association between social distancing and children’s perceived anxiety

There was an association between refraining from social activities and children’s level of anxiety measured by CAQ (Model 1, Table III). Refraining from social activities explained 3.5% (R^2^) of the variance in the dependent variable perceived anxiety. Children who refrained from social activities showed a higher level of perceived anxiety compared with those who did not refrain from social activities during the pandemic. Adjusting for sex and age, chronic diseases/disabilities and reduced income, children who refrained from social activities still showed a significantly higher level of perceived anxiety (Models 2–4, Table III). When the association between refraining from social activities and perceived anxiety was adjusted for all four covariates (Model 4), the beta coefficient decreased from −0.57 to −0.52 and R-square increased from 0.035 to 0.060 (Table III). This indicates that those four background variables together contributed only 6% of the variation in the variable children’s perceived anxiety. Thus, the association between refraining from social activities and children’s perceived anxiety was robust and only marginally influenced by other pandemic-related factors.

### Qualitative results

Out of the 774 participating children, 151 children (89 girls and 62 boys) answered the open question (Table I). The qualitative analysis resulted in four categories: seeing the bright side of life, worrying about others and themselves, missing their loved ones, and feeling limited in their usual activities. Despite the pandemic situation, the children emphasised the bright side of life. The consequences of the pandemic affected their ordinary life, and the children were unable to attend their usual activities. The restrictions became obvious to them and interfered with their relationships.

#### Seeing the bright side of life

The children were able to appreciate the bright side of the situation brought about by the COVID-19 pandemic. The fact that school was still open was seen as positive. ‘I have only been at school, not staying at home. I think it’s good’ (girl 10 years). One aspect of the restrictions was that it meant more time with their parents, which they enjoyed. ‘It’s been fun being home a lot with mom and dad. We have built a lot of Lego‘ (boy 6 years). Furthermore, although they were unable to continue with their usual leisure activities, they appreciated having some close friends they were allowed to meet and play with. ‘Good thing we picked out some friends I can hang out with’ (boy 11 years). The children also emphasised the fact that outdoor activities during school time and breaks increased, which they thought was good. ‘Think it is good to be playing outdoors more at school’ (girl 9 years).

#### Worrying about others and themselves

The children expressed worries about COVID-19 and that it is lethal. However, the children mostly did not indicate that they were worried about their own health, but it was clear that the COVID-19 virus was experienced as frightening. ‘I get scared and worried when I think of Corona’ (girl 13 years). Being afraid of the disease also came up, and they were thinking about how they as children could be affected by COVID-19. ‘Children can be sick for a reeeeaaallllllyyyyyyy long time . . .’ (boy 11 years). More prominent was their worry for their loved ones, their parents and younger siblings, but especially their grandparents, who were seen as being vulnerable. ‘What worries me is that my grandmother or grandfather will get it because they are at a higher risk in more ways than I am’ (girl 7 years). In addition, children expressed altruistic thoughts, feeling responsible to avoid transmitting the virus to other more vulnerable persons or loved ones. ‘I’m careful because I don’t want to infect someone who can infect someone old, because then the old one dies’ (girl 8 years). Furthermore, they were worried that this pandemic might continue for a long time. The children expressed concern about the future. ‘I wonder when the corona will end. I’m worried it’s going to last for years’ (boy 11 years).

#### Missing their loved ones

The pandemic situation meant the absence of people they cherished. The children missed loved ones, often grandmothers and grandfathers, and they missed being able to hug them. ‘I can’t wait to hug my family’ (girl 7 years). They missed their usual social interactions with their loved ones and the things they normally did together, which often made them cry. ‘I cry quite often because I miss them. Mom is crying too, and we are hugging. She says no one knows when it will be over. I hate Corona’ (boy 12 years).

#### Feeling limited in their usual activities

The children expressed not being able to do what they usually do and, because the restrictions limited their activities, they felt disconnected both socially and physically. ‘Boring not to be able to do the same things as usual, as mom and dad want to be careful‘ (boy 12 years). They highlighted the limits on outdoor activities such as sports, competitions and social life with friends, as well as expressing how they missed even their ordinary activities. ‘I miss going to the swimming pool and being at my swimming school. It’s sad that you can’t play sports the same way’ (girl 12 years).

### Integration of qualitative and quantitative results

**Figure 2.**
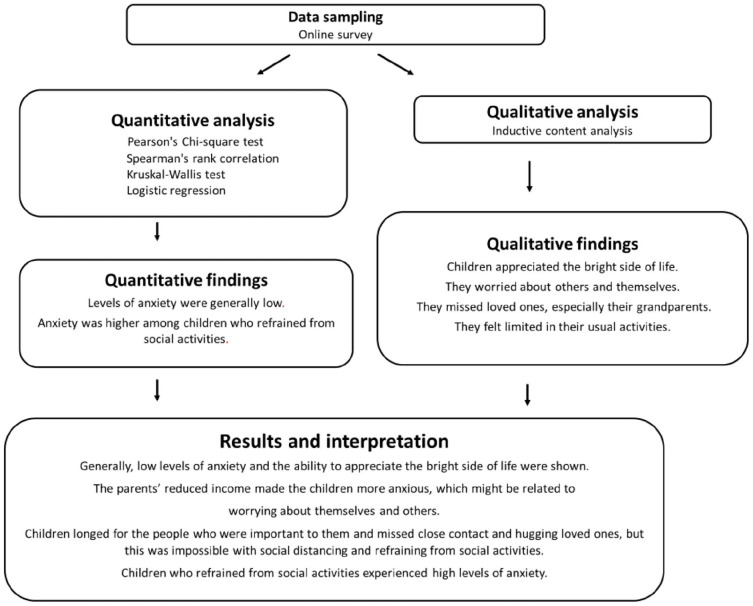
Integration of qualitative and quantitative results.

The integration of the results from the qualitative and quantitative results is shown in Figure 2. Generally, low levels of anxiety were found and an ability to appreciate the bright side of life was shown, even though they were worried about others and missed loved ones, especially their grandparents. Only a few of the children experienced high levels of anxiety, and this was mainly children who were refraining from social activities and children whose parents had a reduced income due to the pandemic. The restrictions imposed due to the pandemic limited the children’s usual activities and their social contact.

## Discussion

The main finding of this study was that most children involved in this Swedish survey experienced low levels of anxiety and an ability to appreciate the bright side of life during the pandemic. At the same time, they felt worried about others and missed loved ones. The year 2020 was a year like no other for many children around the world, and lockdowns and social distancing have been highlighted as important measures to limit the spread of COVID-19 [14]. However, different measures were taken in Sweden compared to many other countries, and the degree of lockdown was lower [16]. For young children, life did not change very much; rather, their everyday contact with their parents increased and, in most cases, they could continue their outdoor activities with their friends.

Anxiety is according to Berde and Wolfe [17] defined as subjective senses of unease and dread and, due to the complexity of measuring anxiety in children, two different visual scales were chosen to capture the children’s emotions. The present study showed that children generally reported low levels of anxiety, with no differences between boys and girls. However, the children who refrained from social activities showed a somewhat higher level of anxiety compared to those who did not refrain. Teens have shown more mental health problems than younger children during the pandemic and the prevalence of depression and anxiety symptoms was higher in girls than boys [18–20].

The effect of school closures as an important measure to minimise the spread of COVID-19 has scarcely been evaluated. However, studies from the severe acute respiratory syndrome (SARS) outbreak in China and Singapore indicate that school closures did not contribute to the control of the epidemic [21]. Despite this, most countries chose to close schools, even though it is known that lockdowns can have a lifelong impact on children’s health [22]. Unlike many other countries, schools for children aged up to 16 years have remained open in Sweden [23]. Children were able to see friends outdoors, which made some aspects of life more normal or, for some children, even better than normal. With the exception of primary school, social distancing was generally recommended in Sweden. In our study, refraining from social activities only explained 6% of the variance in the dependent variable level of experienced anxiety, which could be seen in comparison with the study from Brazil [5], where the children experienced more social distancing and eight times the prevalence of anxiety found in our study.

Children in Sweden have been able to go to school and to meet small groups of friends outdoors, but there could still be an increased risk that children might experience high rates of depression and anxiety. It is therefore necessary for school health staff to increase awareness about anxiety and depression in school-aged children. It is also important to initiate strategies of early prevention of mental illness in order not only to limit but also highlight the risk, especially among children who are more isolated due to the pandemic [24].

In our study, the children expressed worries arising from the pandemic situation; a few were worries for themselves, but mostly the worries were for elderly relatives. In order to reduce the children’s concerns it is important that adults, perhaps above all the schoolteachers, talk to and educate children about COVID-19 in an honest and age-appropriate way to help them deal with their feelings and fears [25,26]. Furthermore, if parents discuss the pandemic situation with their children, there is less risk that the child will experience depression, anxiety and stress [27].

In general, the children in this study reported low levels of anxiety, and most of them could probably cope with their situation. It has been shown that children’s ability to adapt to social distancing restrictions can affect their well-being; for example, by adapting to restrictions and social distancing. Another study, conducted in Chile during the COVID-19 pandemic and lockdown, found that a higher family functioning reduced the likelihood of behavioural and peer problems [28].

The COVID-19 pandemic has had a special impact on the children who have lived through it [9]. To promote a good health and well-being for children the United Nations (UN) Sustainable Development Goals in the 2030 Agenda and WHO and UNICEF have called on the world’s decision-makers to consider the best interests of children [29]. In order to fully understand how children in different countries are affected by the pandemic, there is therefore a need to conduct more long-term studies over the coming years.

## Limitations

A few factors might have influenced the results of this study. The sample of respondents may have been skewed since the survey was initially distributed from the research group’s network; the demographics showed that most of the participants lived in a city with 100,000–499,999 inhabitants, and the guardian was in most cases working, had a university degree and no reduction in income during the pandemic. For the youngest children, the guardian may have influenced the result. The use of convenience sampling meant that we could not systematically ensure that all regions of Sweden were included in the final sample. These factors could be seen as a limitation, since the data does not reflect the diversity of the general population in Sweden. Another limitation might be that we only present data from the first phase of the pandemic and are unable to present data from the following phases.

## Conclusion

Our study showed that most children in our Swedish sample experienced low levels of anxiety, which is in contrast with many other studies. The association between refraining from social activities and level of anxiety was robust and showed that children who refrained from social activities experienced more anxiety. All restrictions were implemented voluntarily in Sweden, where no lockdown was carried out, which might have had an impact on our result. Even though the children experienced the pandemic in some ways as an intrusion on their lives, they also reported positive aspects of the pandemic. Keeping life as normal as possible could be one important factor in preventing higher anxiety and depression levels in children during a pandemic.
